# Botulinum toxin and conservative treatment strategies in people with cervical dystonia: an online survey

**DOI:** 10.1007/s00702-023-02707-5

**Published:** 2023-10-13

**Authors:** Melani J. Boyce, A. B. McCambridge, L. V. Bradnam, C. G. Canning, C. Quel De Oliveira, A. P. Verhagen

**Affiliations:** 1 Discipline of Physiotherapy, Graduate School of Health, University of Technology Sydney, Sydney, Australia; 2 Physiotherapy Department, Westmead Hospital, Hawkesbury Road, Westmead, Sydney, NSW 2145 Australia; 3 Public Health Association of New Zealand, Auckland, New Zealand; 4 Department of Exercise Sciences, The University of Auckland, Auckland, New Zealand; 5 Faculty of Medicine and Health, The University of Sydney, Sydney, Australia

**Keywords:** Cervical dystonia, Survey, Botulinum neurotoxin injections, Physiotherapy

## Abstract

Isolated cervical dystonia is a focal, idiopathic dystonia affecting the neck muscles. Treatment usually consists of botulinum neurotoxin (BoNT) injections into the dystonic muscles. Our aim is to investigate the use of BoNT treatment and conservative treatments by people living with cervical dystonia. An online survey in English was conducted between June and August 2022. Participants were eligible to participate if they were living with cervical dystonia, were over 18 years old and could read and understand English. The survey consisted of demographic questions, characteristics of dystonia, questions relating to BoNT use and the perceived utility of conservative treatments. The data were analysed descriptively, and open-ended questions were grouped into similar topics represented by direct quotes. We received 128 responses from people with cervical dystonia, with an average age of 59 years and 77% women. Most participants (52%) described their cervical dystonia as mild to moderate with an average pain score of 5/10. Eighty-two (64%) participants were having regular BoNT injections, with overall positive perceived effects. Common activities reported to improve the symptoms were the use of heat packs, massage, relaxation, physiotherapy and participation in general exercise. Common coping strategies reported were getting sufficient rest, having the support of friends and family, and remaining engaged in enjoyable hobbies. We found that most participants received regular BoNT injections and that heat packs, exercise, massage, physiotherapy and relaxation were mostly perceived as effective in reducing the symptoms of cervical dystonia.

**Supplementary Information:**

The online version contains supplementary material available at 10.1007/s00702-023-02707-5.

## Introduction

Idiopathic focal isolated cervical dystonia (iCD) is characterised by sustained or intermittent neck movements caused by involuntary muscle contractions, leading to abnormal movements of the head, neck and/or shoulders (Albanese et al. 2023). The neck may turn in any direction, and the dystonia is named accordingly, for example, rotational torticollis (Albanese et al. 2023). Onset is around the age of 40 years and affects women more commonly than men with a ratio of 3:2 (Albanese et al. 2023; ESDE 2000). The primary motor impairment is uncontrollable muscle spasms of the neck; however, at least two-thirds of people with iCD will experience long-term non-motor impairments including headaches and neck pain (Comella and Bhatia 2015). iCD causes significant disability affecting the work, leisure and psycho-social aspects of a person’s life, with 60% of people with dystonia reporting depression and anxiety—a significantly higher rate than the general population (Comella and Bhatia 2015; Demartini et al. 2018; Jahanshahi and Marsden 1990).

The most widely evaluated treatment for iCD is botulinum neurotoxin (BoNT) injections into the dystonic muscles. BoNT injections improve motor and non-motor impairments, reduce pain and improve quality of life (Rodrigues et al. 2020; Contarino et al. 2017). However, its effects wear off and BoNT therapy must be repeated every 3–4 months. Furthermore, the injections are painful and side effects can include dysphagia and excessive muscle weakness (Comella and Thompson 2006). Approximately 20% of people with iCD will discontinue BoNT therapy, with the most frequently reported reasons being the lack of long-term effectiveness and the adverse side effects (Comella and Thompson 2006). Therefore, alternatives to BoNT therapy are highly sought after. However, there are currently no clinical practice guidelines for the management of iCD, meaning that clinicians and people with iCD have no evidence-based guidance for effective, alternative or self-management treatments. Two systematic reviews examined the evidence for conservative management of iCD including physiotherapy, neck exercises, massage, neck mobilisation, manipulation, EMG biofeedback, relaxation and cognitive behavioural therapy. Both reviews concluded that such therapies may improve pain, disability and quality of life for people with iCD in the short-term, however, the quality of the evidence gained from the very few available individual studies was low, and the long-term effects of therapy were under reported (Delnooz et al. 2009; De Pauw et al. 2014).

There is also limited evidence for other conservative treatments for iCD. Acupuncture and chiropractic therapy are yet to be studied in a randomised controlled trial; however, case studies report some benefits (Horibe et al. 2020; Kukurin 2004; Lu et al. 2017; Zhao and Wu 2014). A retrospective survey of 389 people with dystonia in the USA found that 53% of participants had tried at least one conservative treatment, with 48% combining this with BoNT. The study reported that the mean perceived effectiveness of conservative treatments was 28%, versus 59% for the mean perceived effectiveness of BoNT. The most common conservative treatment used by people with iCD was physiotherapy which was undertaken by 27% of participants (Fleming et al. 2012). A survey of 266 German people with iCD found that 81% had tried at least one conservative treatment, with specialised physiotherapy and psychotherapy being rated as the most effective. The least effective conservative treatments reported were massage, chiropractic and acupuncture (Viehmann et al. 2014).

Furthermore, there is little evidence for the use of self-management strategies for people with iCD. One pilot study investigated the feasibility of a 3-day residential program that taught strategies to assist people with iCD to manage their own condition. The training included sessions on goal setting, mindfulness, pain management and sleep, with participants regarding the educational program as beneficial. Pain, anxiety, depression and well-being were scored by participants before and after the program, however this was a feasibility study, and therefore, it was not powered to detect a difference in outcomes (Sandhu et al. 2016).

The full extent and utility of self-management and conservative treatments employed by people with iCD is not yet known. This information is vital to develop guidelines for the treatment of iCD. Therefore, the aim of this study was to investigate the use of BoNT treatment and use of conservative management strategies employed by people living with iCD via an online survey.

## Methods

### Study design

A survey written in English was conducted online using Qualtrics software. A printed version of the survey was available for participants who could not complete the survey online. Ethical approval for this study was obtained from the University of Technology (Sydney) Human Research Ethics Committee prior to commencement (No. ETH21-6635).

### Participants

Participants were eligible to join the study if they had idiopathic, focal iCD diagnosed by a neurologist, were aged over 18 years and were able to speak and read English. The survey was targeted at participants with iCD worldwide. The survey was advertised through the Dystonia Network of Australia, the Australian Dystonia Facebook group, the New Zealand Dystonia Patient Network and Dystonia Europe. The survey was available to complete online between June and August 2022. Participants completed a consent form online prior to commencing the study and were not able to access the survey without first providing consent.

### Data collection

Demographic data consisting of gender, age, country of birth and cultural background were collected. Cultural background was an open question allowing participants to self-describe their cultural identity. The survey contained questions about the characteristics of the participants’ dystonia and included the perceived rating of muscle spasms (mild, moderate and severe) and a pain intensity numerical rating scale that ranged from zero to 10, where zero was absence of pain and 10, the worst pain possible. Questions were asked about participants’ current and previous use of BoNT injections and the perceived utility of BoNT in managing their iCD. In addition, participants were asked to list other therapies or activities that they found helpful in managing their iCD and to rate the usefulness of each in managing pain, muscle spasms and active movement. Answering all questions was not compulsory; participants were able to skip questions if they preferred not to answer. Participation and data collection were anonymous. A complete list of survey questions can be found in the Online resource (Appendix 1).

### Data analysis

Demographic data were analysed descriptively to determine means, standard deviations (SDs), frequencies and ranges. Questions regarding the use of BoNT injections and other therapies were analysed descriptively for each question by calculating the proportion of responses for each answer in relation to the total number of responses. Answers to open-ended questions were interpreted by grouping similar topics. Some quotes written by participants were selected to illustrate the most frequently reported topics.

## Results

### Participants

There were 134 responses; six participants were excluded from the analysis (four consented to participate but recorded no further answers and two participants reported that they did not have iCD). The survey was completed by 128 people with iCD, with an average age of 59 years (SD 12; range 22–89 years), 77% (*n* = 97) of whom were women. All surveys were completed online, and we received no requests for a paper copy of the questionnaire. Ten percent of participants reported having an additional neurological condition. Most participants lived in Australia or New Zealand (96.8%); other participants lived in the UK (1.6%) and the USA (1.6%). Participants were culturally diverse reporting backgrounds from Europe, Australia, New Zealand, Asia and America. Forty-three percent of participants were employed in full or part-time paid work. Most participants (52.4%) reported that their muscle spasms due to iCD were mild or moderate, and the average reported pain score was 5.1 out of 10 (SD 2.9; range 0–10) (Table 1). The full table including all results from the survey is available in the Online resource (Appendix 2).

**Table 1:** Demographics—128 responses

Items (number of respondents)	Answers, *n* (%)
Gender, female (*n* = 126)	97 (77%)
Coexisting neurological condition, yes (*n* = 126)	13 (10.3%)
Average age in years (SD; range) (*n* = 124)	59 (12; 22–89)
Country of residence (*n* = 124)	Australia: 107 (86.3%)
	New Zealand: 13 (10.5%)
	UK/USA: 4 (3.2%)
Cultural background (*n* = 125)	Australian–68 (54.4%)
	European–30 (24%)
	Caucasian–8 (6.4%)
	Asian–4 (3.2%)
	Combined: 13 (10.4%)
	Other: 2 (1.6%)
Time since diagnosis (years) (*n* = 126)	< 1–8 (6.3%)
	2–5–34 (27%)
	6–10–32 (25.4%)
	> 10–52 (41.3%)
Currently employed in paid work (full or part time), yes (*n* = 121)	52 (43%)
Intensity of muscle spasms due to iCD (*n* = 124)	None: 9 (7.3%)
	Very mild/mild: 35 (28.2%)
	Moderate: 52 (41.9%)
	Severe/very severe: 28 (22.6%)
Average pain scored from 0–10 (SD; range) (*n* = 123)	5.1 (2.9; 0–10)
Difficulty of neck movement (*n* = 125)	None: 9 (7.2%)
	Slight: 37 (29.6%)
	Moderate: 56 (44.8%)
	Severe/very severe: 23 (18.4%)

### Botulinum neurotoxin use

Most participants (64%) reported having regular BoNT injections, with 86% of these reporting experiencing therapeutic benefits from them. Participants reported an average of 60% (SD 28%) improvement following injections with a range of 5–100%. Of those having regular BoNT injections, most (68%) had injections every 3 months, 18% had injections more frequently than 3 months, and 14% had an injection interval longer than 3 months. Seventy-six percent of participants were satisfied with their injection intervals. Of those participants dissatisfied with their injection intervals, 61% reported that injection intervals were fixed at a certain time and all reported they would prefer if their injections intervals could be more flexible. The most common reasons for ceasing injections were ineffectiveness of treatment (35%), the negative side effects (21%), the cost (9%) and the effect not lasting long enough (7%). Other less commonly reported reasons for ceasing BoNT included difficulty accessing the clinic (5%), the pain or stress of injections (5%), concern over the inexperience of the doctor administering the injections (2%) and the development of another condition affected by BoNT (2%) (Online resource, Appendix 2).

### Conservative treatments

The perceived effect of various conservative treatments on participants' pain, muscle spasms and ease of neck movement is shown in Tables 2, 3 and 4. Specific physiotherapy treatment approaches were not assessed individually—the term “physiotherapy” referred to any treatment administered by a physiotherapist. The self-perceived beneficial strategies for reducing pain were the use of heat packs, relaxation, medication and massage, reported as useful by more than 40% of participants. Physiotherapy, general exercise, mindfulness and meditation were reported as the next most useful strategies in reducing pain by 30–40% of participants. Muscle spasms were reduced most frequently by using heat packs, massage, medication, relaxation and physiotherapy and were reported by 30–37% of participants. General exercise and mindfulness/meditation were reported as beneficial by 20–30% of participants in reducing muscle spasms. Ease of neck movement was improved after massage, heat packs, physiotherapy, general exercise and medication in 30–40% of participants. Relaxation was the next most useful strategy to ease neck movement, reported by 27% of participants (Tables 2, 3, 4).

**Table 2:** Treatment effect on pain

Intervention	Improved, *n* (%)	No change, *n* (%)	Worsened, *n* (%)	Total, *n* (%)
Heat pack	72 (56.3%)	9 (7%)	4 (3.1%)	85 (66.4%)
Relaxation	57 (44.5%)	9 (7%)	1 (0.8%)	67 (52.3%)
Oral medication	55 (43%)	20 (15.6%)	3 (2.3%)	78 (60.9%)
Massage	53 (41.4%)	16 (12.5%)	18 (14.1%)	87 (68%)
Physiotherapy (incl stretches)	46 (35.9%)	21 (16.4%)	17 (13.3%)	84 (65.6%)
General exercise/sport	41 (32%)	31 (24.2%)	20 (15.6%)	92 (71.9%)
Meditation/mindfulness	39 (30.5%)	13 (10.2%)	2 (1.6%)	54 (42.2%)
Yoga	22 (17.2%)	9 (7%)	13 (10.2%)	44 (34.4%)
Neck collar/brace	20 (15.6%)	11 (8.6%)	5 (3.9%)	36 (28.1%)
Acupuncture/dry needling	18 (14.1%)	23 (18%)	3 (2.3%)	44 (34.4%)
Psychology	15 (11.7%)	13 (10.2%)	1 (0.8%)	29 (22.7%)
Electrical stimulation	12 (9.4%)	11 (8.6%)	6 (4.7%)	29 (22.7%)
Osteopathy	12 (9.4%)	7 (5.5%)	4 (3.1%)	23 (18%)
Naturopathy/herbal remedies	10 (7.8%)	13 (10.2%)	0 (0%)	23 (18%)
Chiropractic	10 (7.8%)	15 (11.7%)	10 (7.8%)	35 (27.3%)
Dietician/specific diet	9 (7%)	9 (7%)	0 (0%)	17 (13.3%)
Farias technique	8 (6.3%)	9 (7%)	2 (1.6%)	19 (14.8%)
Other	9 (7%)	3 (2.3%)	0 (0%)	12 (9.4%)

**Table 3:** Treatment effect on muscle spasm

Intervention	Improved, *n* (%)	No change, *n* (%)	Worsened, *n* (%)	Total, *n* (%)
Heat pack	48 (37.5%)	18 (14.1%)	3 (2.3%)	68 (53.1%)
Massage	48 (37.5%)	16 (12.5%)	16 (12.5%)	80 (62.5%)
Oral medication	48 (37.5%)	30 (23.4%)	4 (3.1%)	82 (64.1%)
Relaxation	48 (37.5%)	12 (9.4%)	2 (1.6%)	62 (48.4%)
Physiotherapy (incl stretches)	39 (30.5%)	23 (18%)	17 (13.3%)	79 (61.7%)
General exercise/sport	36 (28.1%)	24 (18.8%)	21 (16.4%)	80 (62.5%)
Meditation/mindfulness	28 (21.9%)	12 (9.4%)	2 (1.6%)	42 (32.8%)
Yoga	15 (11.7%)	5 (3.9%)	11 (8.6%)	31 (24.2%)
Neck collar/brace	15 (11.7%)	12 (9.4%)	4 (3.1%)	31 (24.2%)
Acupuncture/dry needling	14 (10.9%)	21 (16.4%)	6 (4.7%)	41 (32%)
Naturopathy/herbal remedies	11 (8.6%)	9 (7%)	0 (0%)	20 (15.6%)
Chiropractic	9 (7%)	12 (9.4%)	6 (4.7%)	27 (21.1%)
Electrical stimulation	9 (7%)	8 (6.3%)	6 (4.7%)	23 (18%)
Farias technique	8 (6.3%)	5 (3.9%)	1 (0.8%)	14 (10.9%)
Psychology	7 (5.5%)	14 (10.9%)	0 (0%)	21 (16.4%)
Osteopathy	7 (5.5%)	8 (6.3%)	4 (3.1%)	19 (14.8%)
Dietician/specific diet	6 (4.7%)	7 (5.5%)	2 (1.6%)	15 (11.7%)
Other	5 (3.9%)	2 (1.6%)	0 (0%)	7 (5.5%)

**Table 4:** Treatment effect on ease of movement

Intervention	Improved, *n* (%)	No change, *n* (%)	Worsened, *n* (%)	Total, *n* (%)
Massage	51 (39.8%)	15 (11.7%)	11 (8.6%)	77 (60.2%)
Heat pack	49 (38.3%)	20 (15.6%)	3 (2.3%)	72 (56.3%)
Physiotherapy (incl stretches)	47 (36.7%)	18 (14.1%)	10 (7.8%)	75 (58.6%)
General exercise/sport	41 (32%)	24 (18.8%)	15 (11.7%)	80 (62.5%)
Oral medication	40 (31.3%)	39 (30.5%)	0 (0%)	79 (61.7%)
Relaxation	34 (26.6%)	19 (14.8%)	1(1.9%)	54 (42.2%)
Meditation/mindfulness	23 (18%)	18 (14.1%)	0 (0%)	41 (32%)
Yoga	21 (16.4%)	6 (4.7%)	8 (6.3%)	35 (27.3%)
Acupuncture/dry needling	12 (9.4%)	23 (18%)	1 (0.8%)	36 (28.1%)
Chiropractic	11 (8.6%)	11 (8.6%)	4 (3.1%)	26 (20.3%)
Neck collar/brace	9 (7%)	13 (10.2%)	6 (4.7%)	28 (21.9%)
Electrical stimulation	9 (7%)	8 (6.3%)	5 (3.9%)	22 (17.2%)
Osteopathy	9 (7%)	5 (3.9%)	5 (3.9%)	19 (14.8%)
Farias Technique	9 (7%)	4 (3.1%)	0 (0%)	13 (10.2%)
Psychology	8 (6.3%)	15 (11.7%)	0 (0%)	23 (18%)
Naturopathy/herbal remedies	7 (5.5%)	11 (8.6%)	0 (0%)	18 (14.1%)
Other (specify)	7 (5.5%)	2 (1.6%)	0 (0%)	9 (7%)

Yoga and chiropractic treatment were tried by up to 30% of participants. Yoga was reported as useful by around half of those who had tried it, while chiropractic treatment was reported by the majority to have a minimal or negative effect on their iCD symptoms. Likewise, electrical stimulation, psychology, naturopathy, osteopathy and the Farias technique were tried by fewer than 20% of participants, with half reporting the benefits of psychology for pain and more than half reporting the benefits of the Farias technique and naturopathy in reducing muscle spasms and easing neck movement. The most common treatments reported as detrimental were general exercise, physiotherapy and massage reported by up to 10–16% of participants. Strategies that were tried by less than 5 participants (4%) were included in the “others” section (Tables 2, 3, 4).

### Coping strategies

A third of participants (33%) reported that they were severely affected by iCD, such that they could not perform normal everyday activities, and 61.9% reported slight or great difficulty coping with everyday life. Nearly three quarters of participants (71.7%) reported that it remains difficult or gets more difficult to cope with iCD over time, while only 22.8% reported that it is getting easier over time. Common difficulties reported included driving, socialising and being in stressful situations, for example in the workplace. Four participants described the variability of the condition and the effect that it has on their ability to cope, for example:

“Having had the cervical dystonia for so long it has gone through many stages regarding pain, shaking of the head, ability to cope with everyday life so no one answer applies to my situation”.

Eleven participants described the life modifications that they use to cope with iCD, including the use of medication and making modifications to daily tasks to accommodate the iCD, for example:

“My family and I have changed our whole way of living to cope with my dystonia. So initially it was extremely difficult, now it is ok to cope with”.

**Fig. 1:**
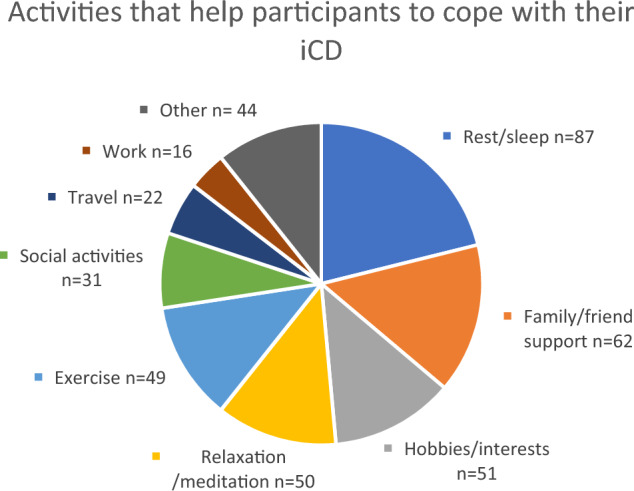
Activities that help participants to cope with their iCD

The most frequently reported strategy to cope with iCD was getting sufficient sleep or rest (*n* = 87). Other commonly reported activities were having the support of family and friends (*n* = 62), being engaged in enjoyable hobbies (*n* = 51), relaxation, mindfulness (*n* = 50) and exercise (*n* = 49) (Fig. 1). Other strategies for coping reported by fewer participants included attending social activities (*n* = 31), travel (*n* = 22), work (*n* = 16), medication (*n* = 11), gardening (*n* = 6), massage (*n* = 3), yoga (*n* = 3) and being in a support group (*n* = 2). One participant described their life modifications to cope with their iCD as “changing my life to reduce stress and physical work as much as possible”, while another participant summarised their condition as “my dystonia is very much affected by what is happening in my life at certain times. This includes both physical and mental situations”.

Forty-six percent of participants (*n* = 53) reported that they would like to have more choices in how they manage their iCD, while 37% (*n* = 43) were unsure. Participants commented that they would like more treatment options, easier access to medical specialists and increased research to find new effective treatments. One participant summarised their desire with this quote “Would like specialist team to help me have a systematic and tested regime effective in managing dystonia”.

Fifty-six percent of participants reported that they took most responsibility for their iCD management, with a further 34% reporting that they relied on their neurologist to manage their dystonia. Two participants described the benefits of having a team approach to their iCD management with these quotes: “The support of my neurologist is also extremely important. I view the management of my Dystonia as like an equal partnership between treatments I have initiated myself and the Botox injections I receive” and “My neuro and I [are] working together to get the best possible outcome”.

## Discussion

We found that despite most participants describing their dystonia as mild or moderate, the majority reported that iCD had a significant negative effect on their ability to engage in normal everyday activities. Most participants were having regular BoNT therapy, with most of them experiencing benefits from the injections and being satisfied with their injection interval. A variety of therapeutic strategies had been tried by our participants, with heat packs, massage, physiotherapy, general exercise and relaxation perceived to be effective at reducing pain and muscle spasms. Participants reported that the best ways to cope with iCD included getting enough sleep, maintaining interests or hobbies, having the support of friends and family, and keeping physically active.

### Comparison with other studies

The self-perceived effectiveness of conservative treatments investigated in our survey was not consistent with some previous studies in people living with iCD (Comella and Bhatia  2015; Fleming et al. 2012; Junker et al. 2004; McCambridge et al. 2019; Viehmann et al. 2014). Participants in a large international survey reported the most effective conservative treatment strategies to be rest, physiotherapy, light exercise and massage, in line with our findings (Comella and Bhatia 2015). Likewise, 80% of the participants in a mailed survey conducted in Germany reported that physiotherapy was effective; however, less than 50% reported that massage, relaxation and manual therapy were effective (Junker et al. 2004). Exercise was self-reported as useful in iCD in the large scale international survey and a smaller survey conducted in the USA (Comella and Bhatia 2015; Fleming et al. 2012), which agrees with our results. However, exercise has also been self-reported to increase the pain, spasms, tremor and fatigue of dystonia in an international online survey (McCambridge et al. 2019), indicating that the response of individuals may differ for unknown reasons that require further research. Massage has also yielded conflicting results, being perceived as effective by participants in our study and other survey studies (Comella and Bhatia 2015; Fleming et al. 2012) but was reported as having minimal or no effect on iCD symptoms by different researchers (Viehmann et al. 2014). The perceived effect of acupuncture and chiropractic treatments was also variable with a reported average effectiveness of 27% (*n* = 20) in the questionnaire study conducted in the USA (Fleming et al. 2012), but either no effect or detrimental effects in people with dystonia in another survey conducted in Germany (Viehmann et al. 2014). As all of these studies were survey designs rating the self-perceived effectiveness of conservative treatments for iCD, it may be that the inconsistent results simply reflect inter-individual differences in perceived effect, personal preferences or the availability and quality of conservative treatments in different geographical locations. Clinical trials are urgently needed to assess the effectiveness of conservative treatments in a methodical and unbiased way to provide definitive answers.

Most of our participants reported that coping with iCD daily either became harder or did not change over time. Coping strategies have not been widely examined in iCD yet, but one survey of people with iCD (O’Connor et al. 2023) found that the types of coping strategies they employed were related to their health-related quality of life and psychological outcomes. Substance use, problem-focussed or avoidance-focussed strategies were found to lead to poorer psychological outcomes and quality of life in people with iCD (O’Connor et al. 2023). Therefore, an approach to treatment that includes positive and healthy coping mechanisms, daily habits and management of psychological health would appear to be vital to improving health-related quality of life. Sleep disturbance is another non-motor symptom of iCD associated with poorer health-related quality of life (Bradnam et al. 2020; Han et al. 2020; Hertenstein et al. 2016; Liang et al. 2022); therefore, sleep management is another intervention strategy that should be incorporated into iCD management by health professionals.

Considering that “Self-management” is the term given to “the individual’s ability to manage the symptoms, treatment and psychosocial consequences and lifestyle changes inherent in living with a chronic condition” (Barlow et al. 2002), including a self-management approach in therapy could potentially improve the quality of life of people with iCD. For example, our participants reported that getting sufficient rest or sleep and being engaged in diversional activities such as socialising with friends and family, having hobbies, performing regular exercise and working were important coping strategies for iCD. These activities are mostly free, can be self-administered and would complement the use of BoNT and other established conservative treatments for iCD (Zetterberg et al. 2018). Future research could focus on the efficacy of a holistic approach to managing iCD including the use of BoNT, conservative adjunct treatments, the support of a team of relevant health professionals and the self-management strategies employed by people with iCD. This approach to managing iCD may result in the best health-related quality of life and psychological outcomes for people with iCD. Feasibility pilot studies are required, followed by robust clinical trials to answer these questions.

### Limitations

The current survey results are not proof of the effectiveness of conservative treatments or self-management strategies for iCD. However, considering the absence of clinical trials assessing conservative treatments for iCD, surveys are important to understand the needs and expectations of this population, in order to develop better management strategies and guide future research.

Our sample size was smaller than previous surveys of people with dystonia (Comella and Bhatia 2015; Fleming et al. 2012; McCambridge et al. 2019; Viehmann et al. 2014), despite our efforts to advertise to participants across the world. Most participants resided in Australia or New Zealand, making the results difficult to generalise to other parts of the world. However, the cultural background of our participants was diverse and reflective of the population of Australia and New Zealand. Future research should endeavour to include participants from across the world to enable generalisability of the results.

Our survey did not specifically ask participants which health professional had diagnosed their iCD, but rather the time since the diagnosis was provided by a neurologist. It is possible that some participants may not have been diagnosed by a neurologist or a movement disorder specialist and so could have “self-identified” as having iCD. Ten percent of participants reported having a neurological condition in addition to iCD, indicating that these participants may not have iCD and possibly cervical dystonia was due to another cause. These limitations may have had a small impact on the results.

### Clinical implications

In the absence of evidence-based conservative treatments and self-management guidelines for managing iCD, this study provides insights into the medical, conservative and self-management treatments that have been undertaken by a cohort of people with iCD, providing subjective reports of their potential effectiveness. This information should inform future research involving people with iCD, with the aim of developing an evidence-based guideline for the management and self-management of iCD to be tested in a clinical trial. As iCD is a rare condition, it is important to have guidelines for management. This would mean that physiotherapists who are not frequently managing people with iCD can provide the most effective treatments and advice on self-management strategies to improve their patient outcomes. Information from our study can be used to inform future research into conservative treatments, either alone or in conjunction with medical treatments, with the aim to devise holistic guidelines for the management and self-management of iCD. In turn, this may improve health and quality of life outcomes of people with iCD.

## Conclusion

Most participants were having regular BoNT therapy, with 86% of these experiencing benefits from the injections. Studies have shown people with iCD have tried numerous conservative treatments for their condition in conjunction with BoNT therapy. Our survey participants reported that the most effective treatments were heat packs, exercise, massage, physiotherapy and relaxation. This understanding adds to the body of knowledge of potential therapeutic interventions and self-management strategies for this recalcitrant movement disorder.

### Supplementary Information

Below is the link to the electronic supplementary material.

Supplementary file1 (PDF 294 kb)

Supplementary file2 (PDF 314 kb)

## Data Availability

All data supporting the findings of this study are available within the paper and its Supplementary Information.
