# Supplementary material for: Botulinum toxin and conservative treatment strategies in people with cervical dystonia: an online survey
Source: J Neural Transm (Vienna). 2023 Oct 13;131(1):43–51. doi: 10.1007/s00702-023-02707-5 (PMC10770185; doi:10.1007/s00702-023-02707-5)
Supplement: Supplementary file 1 — Supplementary file1 (PDF 294 kb) [file 702_2023_2707_MOESM1_ESM.pdf]

Appendix 1. Botulinum toxin and conservative treatment strategies in people with cervical dystonia; an online survey

Journal of Neural Transmission

This survey is designed to gather the opinion of people with cervical dystonia on common and uncommon therapies used to manage their dystonia. We would like to assess if there are therapies or activities that are helpful to people with cervical dystonia that fall outside the realm of usual medical care. This information may be useful to others with cervical dystonia, especially those newly diagnosed with the condition.

Please answer the questions below.

Question 1: Do you have cervical dystonia, that is, dystonia that affects your head or neck region?

- yes

- no Please go to the end of the survey

Question 2: Do you have any other neurological conditions, for example, stroke, brain injury, Parkinson's Disease?

- yes Please go to the end of the survey

- no

Question 3: Please indicate your gender.

- Male

- Female

- Non-binary

- Prefer to self-describe

Question 4: Please indicate your current age.

Question 5: Please record the country where you live.

Question 6: Please record your cultural background.

Question 7: How would you rate the severity of your PAIN due to cervical dystonia?

- I don't have any pain due to cervical dystonia

- My pain is very mild

- My pain is mild

- My pain is moderate

- My pain is severe

- My pain is extremely severe

Question 8: How would you rate the severity of your MUSCLE SPASMS due to cervical dystonia?

- I don't have any muscle spasms due to cervical dystonia

- My muscle spasms are very mild

- My muscle spasms are mild

- My muscle spasms are moderate

- My muscle spasms are severe

- My muscle spasms are extremely severe

Question 9: How difficult is it for you to turn your head in all directions?

- I don't have any difficulty turning my head in all directions

- It is slightly difficult to turn my head in all directions

- It is moderately difficult to turn my head in all directions

- It is severely difficult to turn my head in all directions

- It is impossible to turn my head in all directions

Question 10: How much does your cervical dystonia affect the performance of your usual daily activities?

- not at all, my dystonia does not affect me

- my dystonia affects my ability to do my usual daily activities a little

- my dystonia affects my ability to do my usual daily activities a lot

- I am unable to do my usual daily activities because of my dystonia

Question 11: Are you currently in paid employment?

- yes, full time paid employment

- yes, part time paid employment

- no, I retired because of my cervical dystonia

- no, I retired for other reasons

- no, I have never been in paid employment

Question 12: How many years has it been since you were diagnosed with cervical dystonia by a Neurologist?

- less than 1 year

- 2 – 5 years

- 6 - 10 years

- > 10 years

Question 13: How are you coping with your cervical dystonia?

- I find it very difficult to cope with my cervical dystonia

- I find it slightly difficult to cope with my cervical dystonia

- I am unsure how well I cope with my cervical dystonia

- I find it easy to cope with my cervical dystonia

- I find it very easy to cope with my cervical dystonia

- other (please specify)

Question 14: Do you find it easier or harder to cope with your cervical dystonia as time goes on?

- Easier

- Harder

- Unsure

Question 15: What things have helped you to cope better with your cervical dystonia?

- support of family and friends

- work

- social activities

- enjoyable hobbies

- exercise

- rest/relaxation

- travel

- other – please specify

Question 16: Do you currently receive Botulinum toxin (BoNT) injections to treat your cervical dystonia?

- Yes if 'yes', go to Question 20

- No if 'no', go to Question 17

Question 17: Have you tried Botulinum toxin injections for your cervical dystonia in the past?

- Yes if 'yes', go to question 19

- No if 'no', go to question 18

Question 18: If you have NEVER tried Botulinum toxin injections for your cervical dystonia, why not? (tick all relevant answers)

- I don't like needles or having injections

- I am concerned about the use of toxin in my body

- I am concerned about the side effects of Botulinum toxin

- I can't get to the clinic that provides Botulinum toxin injections (e.g. clinic is too far away, open at inconvenient times, no one can help me get there etc.)

- The injections are too expensive

- I don't need Botulinum toxin injections to manage my cervical dystonia

- I have another medical condition which can be affected by Botulinum toxin injections

- I am using a different treatment

- My doctor has never offered me Botulinum toxin injections as a treatment option

- Other (please specify)

Please go to question 27.

Question 19: If you have tried Botulinum toxin injections in the past and STOPPED, why did you stop? (tick all relevant answers)

- Injections didn't relieve my symptoms / didn't work for me

- The side effects were unbearable

- The benefit of injections didn't last long enough

- It was too difficult/inconvenient to get to the injection appointments (for example, the clinic was too far away; I couldn't take time off work; I was caring for others etc.)

- Injections were too expensive to continue long term

- Injections were too painful or stressful to repeat every 3 months

- The doctor wasn't experienced in administering the injections

- I developed another medical condition that would have been affected by continuing Botulinum toxin injections

- Other (please specify)

Please go to question 27.

Question 20: Do you feel that the Botulinum toxin injections are helpful in managing the symptoms of your cervical dystonia?

- yes

- no

- unsure

- **if 'yes',** how much better do you feel after your injection? (Please express you answer in a percentage): ________ % better

Question 21: How affordable are the botulinum toxin injections?

- I do not pay for the injections

- I find the injections are not expensive, I can easily afford them

- I find the injections expensive but I can afford them

- I find the injections are very expensive and I struggle to afford them

- I find the injections are too expensive and I cannot afford to have them

Question 22: How often do you receive injections?

- less than 3 monthly

- every 3 months

- every 4-6 months

- every 6- 12 months

- every 1-2 years or longer

Question 23: Are you satisfied with this injection interval?

- Yes if 'yes', go to question 27

- No if 'no', go to question 24

Question 24: Do you have the option of having the injections at different times, for example, sometimes 3 months apart and sometimes 4 months apart?

- Yes, injection times are flexible and I can book when I need it if 'yes', go to question 27

- No, injection dates are set and are not flexible if 'no', go to question 25

Question 25: Would it be better for you to have the option to have your injections at a different time period?

- Yes if 'yes', go to question 26

- No if 'no', go to question 27

Question 26: How would flexible injection time periods make a difference to you? (tick all relevant answers)

- Flexible injection times would be more convenient for my lifestyle

- Flexible injection times would give me better relief of my cervical dystonia symptoms as I could have the injection when I needed it

- Flexible injection times would help to reduce the side effects of Botulinum toxin

- Flexible injection times would help me financially

- Flexible injection times would be better for my mental health (i.e. reduce my worry/stress)

- other (please specify)

Question 27: What other therapies have you tried at least once to help manage your cervical dystonia? Please tick all that apply and rate what effect each therapy had on the PAIN from your cervical dystonia. Choose from "made my pain better"; "no change in pain" or "made my pain worse".

- I don't have any pain with my cervical dystonia go to question 28

- oral medications

- Physiotherapy, including stretches and neck exercises

- general exercise

- massage
- yoga
- Farias technique
- Chiropractic treatment
- Acupuncture
- Osteopathy
- herbal remedies / Naturopathy
- relaxation
- meditation / mindfulness
- Psychology
- Dietician / specific diet
- Neck collar / brace
- Electrical stimulation
- Technology / Apps
- other (please specify)

Question 28: Please rate the effect each therapy had on the MUSCLE SPASMS from your cervical dystonia**.** Choose from "made my muscle spasms better"; " no change in muscle spasms" or "made my muscle spasms worse".

- I don't have a problem with muscle spasms due to cervical dystonia go to question 29
- oral medications
- Physiotherapy, including stretches and neck exercises
- general exercise
- massage
- yoga
- Farias technique
- Chiropractic
- Acupuncture
- Osteopathy
- herbal remedies / Naturopathy
- relaxation

- meditation / mindfulness
- Psychology
- Dietician / Specific diet
- neck brace / collar
- Electrical stimulation
- Technology / Apps
- other (please specify)

Question 29: Please rate the effect each therapy had on the EASE OF MOVEMENT of your neck and upper body**.** Choose from “made my movement better”; “no change to my movement” or “made my movement worse”.

- I don’t have any problems with moving my neck or upper body go to question 30
- oral medications
- Physiotherapy, including stretches and neck exercises
- general exercise
- massage
- yoga
- Farias technique
- Chiropractic treatment
- Acupuncture
- Osteopathy
- herbal remedies / Naturopathy
- relaxation
- meditation / mindfulness
- Psychology
- Dietician / specific diet
- Neck collar / brace
- Electrical stimulation
- Technology / Apps
- other (please specify)

Question 30: Please tick all activities that you find are the most beneficial in managing your cervical dystonia symptoms.

- oral medications (tablets)
- Physiotherapy, including stretches and neck exercises
- general exercise
- massage
- Farias technique
- Chiropractic
- Acupuncture
- Osteopathy
- herbal remedies / Naturopathy
- relaxation
- meditation / mindfulness
- Psychology
- Dietician / specific diet
- neck brace / collar
- Electrical stimulation
- Technology / Apps
- other (please specify)

Q 31. Please rate the importance you place on each of the following in managing your cervical dystonia. Choose from "very important"; "somewhat important" or "not important".

- Medication (including Botulinum toxin injections)
- Exercise
- Rest / sleep
- Maintaining a healthy diet
- Meditation / Mindfulness
- Social interactions with friends and family
- Continuing previous enjoyable activities (e.g. sports, hobbies, gardening etc.)
- Support of friends and family
- Support of others with cervical dystonia

Question 32: Overall, how satisfied are you with your current cervical dystonia management?

- Very satisfied

- Satisfied

- Unsure

- Unsatisfied

- Very unsatisfied

Question 33: Who is most responsible for managing your cervical dystonia?

- I take the most responsibility for managing my dystonia

- A family member or friend is most responsible for managing my dystonia

- My Neurologist is most responsible for managing my dystonia

- My General Practitioner (GP) is most responsible for managing my dystonia

- Another health professional is most responsible for my dystonia management (please specify)

Question 34: Would you like more choice in how you manage your cervical dystonia into the future?

- yes - please explain what choices you would like

- no

- unsure

Question 35:

If there is anything else you would like to tell us regarding how you manage your cervical dystonia, please write your comments in the box below.

Thank you for taking the time to complete this survey. If you would like any further information regarding this study, or you would like a summary of the results when available, please contact Melani Boyce on email melanijane.boyce@student.uts.edu.au.
