# Supplementary material for: Botulinum toxin and conservative treatment strategies in people with cervical dystonia: an online survey
Source: J Neural Transm (Vienna). 2023 Oct 13;131(1):43–51. doi: 10.1007/s00702-023-02707-5 (PMC10770185; doi:10.1007/s00702-023-02707-5)
Supplement: Supplementary file 2 — Supplementary file2 (PDF 314 kb) [file 702_2023_2707_MOESM2_ESM.pdf]

Appendix 2. Complete Survey Results

Botulinum toxin and conservative treatment strategies in people with cervical dystonia; an online survey

Journal of Neural Transmission

Number of responses – 134; Consent given by 134 participants

4 participants recorded no further answers -> excluded from analysis

2 participants denied having CD -> excluded from analysis

Result n = 128;

Note: not all questions were answered by all participants

Note: the term "Botox*" reported by participants in their comments refers to "Botulinum Neurotoxin" or BoNT

Q 1 Do you have CD? (130 responses)	Yes – 128<br>No - 2
Q2 Do you have any other neurological conditions, for example, stroke, brain injury, Parkinson's Disease? (126 responses)	Yes – 13 (10.3%)<br>No – 113 (89.7%)
Q3 Gender (126 responses)	Female – 97 (77.0%)<br>Male – 29 (23%)<br>Non-binary – 0<br>Prefer to self-describe - 0
Q4 – Age (124 responses)	Range – 22 – 89 years<br>Average – 58.9 years
Q5 Country of residence (124 responses)	Australia – 107 (86.3%)<br>New Zealand – 13 (10.5%)<br>USA – 2 (1.6%)<br>UK - 2 (1.6%)
Q6 Cultural background (125 responses)	Australian – 68<br>United Kingdom – 14<br>European – 12<br>Caucasian – 8<br>European / New Zealand – 4<br>Australian / UK/ Irish – 3<br>Australian / Irish – 2<br>New Zealand - 2<br>New Zealand /Australian - 1<br>USA – 1<br>Bangladeshi – 1<br>Welsh – 1<br>Asian – 1

	Māori – 1<br>Irish – 1<br>Scottish – 1<br>Turkish – 1<br>Chinese / Malay – 1<br>English / Irish – 1<br>English / Australian / Vietnamese - 1
Q7 How would you rate the severity of your PAIN due to cervical dystonia on a scale of 0 - 10? (123 responses)	Range – 0 – 10<br>Average – 5.1
Q8 How would you rate the severity of your MUSCLE SPASMS due to cervical dystonia? (124 responses)	None – 9 (7.2%)<br>Very mild – 13 (10.5%)<br>Mild – 22 (15.5%)<br>Moderate – 52 (41.9%)<br>Severe – 26 (21.0%)<br>Extremely severe – 2 (1.6%)
Q9 How difficult is it for you to turn your head in all directions? (125 responses)	Not difficult at all – 9 (7.2%)<br>Slightly difficult – 37 (29.6%)<br>Moderately difficult – 56 (44.8%)<br>Severely difficult – 19 (15.2%)<br>Impossible – 4 (3.2%)
Q10 How much does your cervical dystonia affect the performance of your usual daily activities? (125 responses)	No effect – 5 (4%)<br>A little – 39 (31.2%)<br>Moderate – 48 (38.4%)<br>A lot – 31 (24.8%)<br>Unable to perform daily activities – 2 (1.6%)
Q11 Are you currently in paid employment? (121 responses)	Yes, full time – 18 (14.9%)<br>Yes, part time – 34 (28.1%)<br>No, retired due to CD – 39 (32.2%)<br>No, unemployed for reasons unrelated to CD – 30 (24.8%)
Q12 How many years has it been since you were diagnosed with cervical dystonia by a Neurologist? (126 responses)	Less than 1 – 8 (6.3%)<br>2-5 years – 34 (27%)<br>6 – 10 years – 32 (25.4%)<br>More than 10 years – 52 (41.3%)
Q13 How are you coping with your cervical dystonia? (126 responses)	Very difficult to cope – 31<br>Slightly difficult to cope – 47<br>Unsure – 15<br>Easy to cope – 11<br>Very easy to cope – 0<br>Other – 22<br>Comments:<br>• Having had the Cervical distonia for so long it has gone through many stages regarding pain, shaking of the head,

	ability to cope with everyday life so no one answer applies to my situation<br>• There's particular situations I have it difficult to cope with my CD such as socilasing or driving<br>• I have a fully fused cervical spine due to my dystonia being misdiagnosed. Hence no movement in my neck and often some pain.<br>• It varies from very difficult to mildly difficult<br>• My cervical dystonia impacts on senior work positions, energy levels and personal confidence at times<br>• Severity changes depending on when I am due for Botox*<br>• I am not completely unable to cope with my Dystonia but make 'modification choices' in my daily activities as the symptoms of my disorder allow, some days more severe than others<br>• It's difficult to copy with certain activities such as speaking with people or driving<br>• I am trying to live with it without Botox*.one year no Botox* after 8+ yrs of reg botox*<br>• If I'm stressed I can still perform my normal daily tasks but they are more challenging<br>• Difficult (i.e. between very and slightly). it also varies.<br>• Had a Neurostimulator or Pacemaker installed 2017<br>• My family and I have changed our whole way of living to cope with my dystonia. So initially it was extremely difficult, now it is ok to cope with.<br>• It ia a very variable codition so it ranges throught all the response, they way i have learnt to live with it is by doing what you can when you can.<br>• I am able to go along with the severe limitations which are a

	consequence of my cervical dystonia. I suppose I'd say I am able to, rather than call it easy or difficult.<br>• I used to suffer bad with spasms and pain but since I have had DBS I would say it's about 95% better<br>• I HAVE NDIS WORKERS 7 DAYS A WEEK<br>• It is easier with medications<br>• I have ok days and not so good days<br>• In the first 5 years it was very difficult. Now it is a lot easier because of treatment I have received over the last 12 years.<br>• I am coping reasonably well since I was diagnosed with cervical dystonia after going on medications, but I occasionally have breakthrough pain, spasms, and trouble with the movement of my head.
Q14 Do you find it easier or harder to cope with your cervical dystonia as time goes on? (123 responses)	Easier – 28<br>Harder – 39<br>No change - 48<br>Unsure - 8
Q15 What things have helped you to cope better with your cervical dystonia? (412 responses)	Support of family and friends – 62<br>Work – 16<br>Social activities – 31<br>Hobbies – 51<br>Exercise – 49<br>Rest / sleep – 87<br>Relaxation / Meditation / Mindfulness – 50<br>Travel – 22<br>Other (specify) - 44<br>Comments:<br>• My distonia is very much affected by what is happening in my life at certain times. This includes both physical and mental situations<br>• My symptoms have fortunately reduced in severity over time, even if I temporarily discontinue my Botox* injections<br>• Yoga stretches daily<br>• Botox*

	• Botox* for spasmodic dysphonia<br>• Massage & Chiropractor<br>• Changing my life to reduce stress and physical work as much as possible<br>• Alcohol<br>•<br>• Valuim<br>Botox*<br>my faith in God<br>Breathing exercises, finding information about the disorder and understanding it<br>Distractions help, rest helps, a neck rest helps and Valium a few times a week helps<br>• I find out if I rest too much I feel worse in the long run. I try to stay active and move my body as much as possible<br>• Being in a support group, and participating in research projects, which have both helped me understand what's going on in my body.<br>• Social activities, hobbies and exercise are all things I miss because of my dystonia. I am either too tired or no longer capable. My hobby used to be bush walking.<br>• Gardening. Yoga.<br>• Massage and muscle relaxants eg Xanax or valium<br>• Feldenkrais, yoga, physiotherapist, psychologist<br>• Botox*, physio, cognitive behavioural therapy, meeting and reading about the plight of others with the same condition<br>• Medication, massage,botox*<br>• Things that distract the brain.<br>• Xeomin<br>• Therapies. Physio,massage<br>• BOTOX*<br>• Acceptance<br>• Daily neck stretching; Daily overall body core muscle toning; Dancing; Music; Heat/Ice; Pain killers; Zero gravity chair to rest my head; Taking

	in nature and beautiful scenery; Breathwork; Hand-held percussion vibration machine<br>• Botox* injections every 3 months<br>• Heat packs, Dencorub<br>• Nothing<br>• Pain education<br>• Specific massage and regular chiropratic and stretching excersise
Q16 Do you currently receive Botulinum toxin (BoNT) injections to treat your cervical dystonia? (123 responses)	Yes – 79 (64.2%)<br>No – 44 (35.8%)
Q17 If no, have you tried Botulinum toxin injections for your cervical dystonia in the past? (44 responses)	Yes – 33 (75%)<br>No – 11 (25%)
Q18 If you have NEVER tried Botulinum toxin injections for your cervical dystonia, why not? (18 responses)	Dislike of needles/injections – 2 (11%)<br>Concern using toxin in my body – 3 (16.7%)<br>Concern about side effects of BTN – 1 (5.5%)<br>Clinic inaccessible – 0<br>Injections are too expensive – 1 (5.5%)<br>No need for injections – 5 (27.8%)<br>Presence of a confounding medical condition – 0<br>A different treatment is working well – 2 (11%)<br>Not offered BTN as a treatment option – 0<br>Other (specify) – 4 (22.2%)<br>Comments:<br>• To my surprise, my cervical dystonia has been milder in recent years and I am no longer afraid of it becoming much worse.<br>• I did not want to have my life governed by the intervals between injections<br>• I had Botox* from 1980s through to 1999 when I opted to retire. As it only worked minimally I opted for a neurostimulator ie pacemaker<br>• Trying other ways first
Q 19 If you have tried Botulinum toxin injections in the past and STOPPED, why did you stop? (56 responses)	Injections didn't work (ineffective) – 15 (26.7%)<br>Unbearable side effects – 10 (17.9%)<br>Benefit of BTN didn't last long enough – 4 (7.1%)

	Too difficult to access clinic – 3 (5.4%)<br>Injections too expensive – 5 (8.9%)<br>Injections too painful/stressful – 3 (5.4%)<br>Inexperienced doctor – 1 (1.8%)<br>Development of another medical condition affected by BTN – 1 (1.8%)<br>Other (specify) – 14 (25%)<br>Comments:<br>• Just gave me a stiff neck, did not stop the tremors<br>• It locked my head into a position which created painful cramps in my shoulder and neck<br>• My neurologist left and hard to start again<br>• I experienced hot flashes when I got the Botox* injections<br>• I became immune to the toxin. It stopped working.<br>• My Neck position is much better and i did not receive botox* since Sep-21. This is based on my consultation with Nuero.<br>• I wanted to try and find a natural solution<br>• Exacerbated head drop to extent that I felt like suiciding<br>• They became unnecessary over time<br>• Dysphagia due to botox*<br>• Injections had side effects. Taking time away from work has been a issue in the past<br>• I started Botulinum toxin at the same time as Sinemet but needed to come off Botox* as it was more important for me to have a lifetime of Botox* dosage needed to go higher in the larynx. As it turned out the Sinemet works well enough that I didn't need to have Botox* for the CD.
Q 20 If you are having regular injections, do you feel that the Botulinum toxin (BoNT) injections are helpful in<br>managing the symptoms of your cervical dystonia? (78 responses)	Yes – 66<br>No – 2<br>Unsure – 10<br>If 'yes', by how much:<br>51 responses

	Range = 5 - 100%<br>Average = 60.1%
Q 21 How affordable are the botulinum toxin injections? (79 responses)	No cost – 21<br>Not expensive, can easily afford them – 28<br>Expensive but affordable – 22<br>Very expensive, struggle to afford them – 8<br>Can't afford to have them – 0
Q 22 How often do you receive injections? (79 responses)	Less than 3 monthly – 14 (18%)<br>Every 3 months – 54 (68%)<br>Every 4-6 months – 8 (10%)<br>Every 6-12 months – 1 (1.3%)<br>Every 1-2 years or longer – 1 (1.3%)<br>I don't have regular injections – 1 (1.3%)
Q 23 Are you satisfied with this injection interval? (79 responses)	Yes – 60 (76%)<br>No – 19 (24%)
Q 24 If no, do you have the option of having the injections at different times,<br>for example, sometimes 3 months apart and sometimes 4 months apart? (18 responses)	Yes, flexible injection times – 7<br>No, not flexible injection times - 11
Q 25 If no, would it be better for you to have the option to have your<br>injections at a different time period? (11 responses)	Yes – 11<br>No - 0
Q 26 How would flexible injection time periods make a difference to<br>you? (11 responses)	More convenient – 2<br>Better relief of CD symptoms – 6<br>Reduced side effects – 0<br>Financially better – 0<br>Reduce worry/stress – 1<br>Other (specify) - 2<br>Comments:<br>• If I could get longer relief from injections so less frequent<br>• I would like regular 3 monthly injections, but the public clinic where I attend does not reliably offer this. For instance, I have to wait 17 weeks for my next lot of injections and have no choice.
Q 27 – Q 31 see separate tables	
Q 30 Please tick ALL activities that you find are beneficial in managing<br>your cervical dystonia symptoms (463 responses)	Oral medication – 51<br>Physiotherapy – 41<br>General exercise / sport – 49<br>Massage – 51<br>Heat pack – 61<br>Yoga – 17<br>Farias technique – 8

	Chiropractic – 6<br>Acupuncture – 11<br>Osteopathy – 7<br>Naturopathy / herbal remedies – 11<br>Relaxation – 47<br>Meditation / Mindfulness – 34<br>Psychology – 15<br>Dietician / specific diet – 8<br>Neck collar / brace – 12<br>Electrical stimulation – 10<br>Technology / Apps – 3<br>Other (specify) – 21<br><br>• please note: chiropractic treatment uses Sacro Occipital Technique'<br>• Physiotherapy on the shoulders and rest of body<br>• Karate<br>• Alcohol<br>• Botox* reduces head tremor<br>• Information about the disorder and getting to understand it<br>• Icepack under neck and back fairly regularly<br>• Sleep<br>• Botox*<br>• Pilates<br>• Travel, hiking<br>• Botox*<br>• Feldenkrais method, Gyrotonics<br>• Bowen therapy<br>• Botox*<br>• DBS had helped in all aspects<br>• Pain education<br>• Ultra Muscleze Magnesium Cream<br>• Sleep<br>• Myotherapy<br>• Brain plasticity exercises<br>•
Q 32 How satisfied are you with your current cervical dystonia management? (115 responses)	Very satisfied – 30<br>Somewhat satisfied – 39<br>Neither satisfied or dissatisfied – 18<br>Somewhat dissatisfied – 17<br>Very dissatisfied – 11

Q 33 Who is most responsible for managing your cervical dystonia? (116 responses)	Myself – 65<br>Family member or friend – 0<br>Neurologist – 40<br>General Practitioner – 1<br>Another health professional (specify) – 10<br>Comments:<br>• The support of my neurologist is also extremely important. I view the management of my Dystonia as like an equal partnership between treatments I have initiated myself and the Botox* injections I receive.<br>• Neurologist, pain specialist myself<br>• Speech therapist who kept an eye on my neck as well as my voice and eyelids<br>• Waiting 18 months for MRI will be having June 2022<br>• My neuro and I working together to get the best possible outcome
Q 34 Would you like more choice in how you manage your cervical dystonia into the future? (116 responses)	Yes – 53 (see comments below)<br>No – 20<br>Unsure – 43<br>Comments:<br>• Would like specialist team to help me have a systematic and tested regime effective in managing dystonia.<br>• I would like to know if medicinal cannabis might be beneficial. However, I have trialled it for myself with the support of my GP, but there has been no tangible benefit so far. However the options for dosage regimen and specific medical formulations are way too broad. There should be properly funded research into this.<br>• More options from doctor, easier accessibly to see physicians<br>• I don't know what choices are available<br>• To be taken seriously, by all health workers on what actually helps the most, rather than a 'one fits all' approach or outlook or expectation.

Q 35 Final comments on how you manage your dystonia (optional)	• I have had to completely manage my dystonia. There do not appear to be any experts in the field. Each person I see tells me there is no really effective treatment and it's a matter of trial and error. No neurologist I have seen has ever suggested I see any other therapist. It seems to be generally accepted that because focal dystonia is comparatively rare there is little research into the condition. Professionals in the movement disorder appear to prefer to specialise in the more common movement disorders such Parkinson's disease where they can see many more patients and earn more income.<br>• My distonia has been occurring for so long and I have tried so many different treatments that don't make a difference or make the trembles worse that I just try to live my life the best I can with the distonia and do what I can to help myself depending on the reasons the condition either abates a little, or gets worse at certain times<br>• Last 2 months I have done 3 standing yoga stretches every morning and have noticed a big reduction in the frequency of headaches I normally experience. I attribute the headaches to overuse of shoulder & neck muscles that have been weakened by botox*. Headaches normally start overnight and come from pain in back of my skull and neck.<br>• Overall, I find the best way to manage my pain is to keep occupied. I am retired and find gardening, hobbies etc keep me occupied. This helps greatly to take my mind off the pain.

Q27. Other treatments tried and their effect on PAIN in CD

	A lot better	A little better	No change	A little worse	A lot worse	Total responses
Oral medication	18	36	20	2	1	87
Physiotherapy (incl stretches)	11	35	21	10	7	84
General exercise / sport	11	27	31	8	12	89
Massage	14	37	16	8	10	85
Heat pack	21	51	9	0	4	85
Yoga	10	11	9	4	9	43
Farias Technique	2	6	9	0	2	19
Chiropractic	4	6	15	1	9	35
Acupuncture	5	12	23	2	1	43
Osteopathy	3	9	7	2	2	23
Naturopathy / herbal remedies	3	7	13	0	0	23
Relaxation	18	39	9	0	1	67
Meditation / Mindfulness	12	27	13	1	1	54
Psychology	7	8	13	1	0	29
Dietician / specific diet	3	6	8	0	0	17
Neck collar / brace	4	15	11	3	2	35
Electrical stimulation	6	6	11	3	3	29
Technology / Apps	0	2	7	0	2	11
Other (specify)	9	5	2	0	0	16

Other / Comments:

- Feldenkrais – Made pain a little better (x1)
- Bowen therapy – made pain a little better (x1)
- Feldenkrais – made pain a lot better (x1)
- Medications – made pain a lot better
- Bowen therapy x 1 – no response for effect on pain

- Tai Chi and Bowen therapy – no change to pain (x1)
- DBS – pain a lot better (x1)
- Medicinal cannabis – no change to pain (x1)
- Pain education – made pain a lot better (x1)
- Ultra Muscleeze Magnesium Creame – made pain a lot better (x1)
- Brain plasticity exercises – made pain a lot better
- Soft neck collar (x 1) - Soft neck collar helped with neck drop after Botox* and driving, gardening and when I need to concentrate on the computer, watching tv. etc. These things still cause pain if I spend too much time doing them or I don't take my medication on time.
- Dietician / Speech pathologist - I have been seeing a dietician and on a soft diet for dysphagia since before I was diagnosed with CD symptoms of my dystonias. I can't remember if I had any pain due to it. I am still on a soft diet. (x1)
- Dry needling – made pain a little better (x1)
- Gentle massage with Voltaren emugel or antiflamme cream – made pain a lot better (x1)
- Karate – made pain a lot better (x1)
- Exercise varies - walking is great for example but cycling really hurts neck (x1)
- prayer, exercise, yoga stretches assist brain/ soul wellbeing – made pain a little better (x1)
- Icepack I sleep with them under my neck and back – Made pain a lot better
- Stellate ganglion block to RHS neck – made pain a little better (x1)

Q 28. Effect of each treatment on MUSCLE SPASMS of CD

	A lot better	A little better	No change	A little worse	A lot worse	Total responses
Oral medication	12	35	30	4	0	81
Physiotherapy (incl stretches)	11	28	23	10	7	79
General exercise / sport	11	24	24	13	8	80
Massage	13	34	16	10	6	79
Heat pack	14	34	18	0	3	69
Yoga	9	6	5	5	6	31
Farias Technique	2	6	5	0	1	14
Chiropractic	4	5	12	2	4	27
Acupuncture	4	9	21	5	1	40
Osteopathy	2	5	8	0	4	19

Naturopathy / herbal remedies	2	9	9	0	0	20
Relaxation	11	37	12	0	2	62
Meditation / Mindfulness	7	21	12	1	1	42
Psychology	3	4	14	0	0	21
Dietician / specific diet	1	5	7	2	0	15
Neck collar / brace	2	13	12	2	2	31
Electrical stimulation	3	6	8	2	4	23
Technology / Apps	1	1	5	0	2	9
Other (specify)	4	3	1	0	0	8

Other / comments:

- Bowen therapy – made spasms a little better
- Feldenkrais – a lot better
- Tai Chi and Bowen therapy – no change to muscle spasms
- DBS
- Endone – made spasms a little better
- Ultra muscleze
- Brain plasticity exercises – made spasms a lot better
- Dry needling – made spasms a little better
- Karate – made spasms a lot better
- Icepack under head and neck – made spasms a lot better

Q 29 Effect of each treatment on EASE OF MOVEMENT in CD

	A lot better	A little better	No change	A little worse	A lot worse	Total responses
Oral medication	13	27	39	0	0	79
Physiotherapy (incl stretches)	15	32	18	6	4	75
General exercise / sport	9	31	24	9	6	79

Massage	13	37	15	7	4	76
Heat pack	11	37	20	0	3	71
Yoga	7	14	6	2	6	35
Farias Technique	3	6	4	0	0	13
Chiropractic	7	4	11	0	4	26
Acupuncture	3	8	23	1	0	35
Osteopathy	4	5	5	2	3	19
Naturopathy / herbal remedies	1	6	11	0	0	18
Relaxation	5	29	19	0	1	54
Meditation / Mindfulness	5	17	18	0	0	40
Psychology	3	5	15	0	0	23
Dietician / specific diet	0	4	8	0	0	12
Neck collar / brace	2	7	13	2	3	27
Electrical stimulation	4	5	8	2	3	22
Technology / Apps	1	0	6	0	2	9
Other (specify)	6	4	1	0	0	11

Other / Comments:

- Dry needling – made movement a little easier
- Karate – made movement a lot easier
- Neck collar – weakened my neck strength
- Icepack under neck and back – made movement a lot easier
- Bowen therapy – made movement a little easier (x 2)
- Feldenkrais – made movement a lot easier
- Brain plasticity exercises – made movement a lot easier
- Myotherapy – made movement a little easier
- Ultra Muscleze Magnesium – made movement a lot easier
- DBS – made movement a lot easier
- Tai Chi and Bowen therapy – no change to movement
-
- If needed I also use heated rice bags on my neck for cervical dystonia. I'm able to walk on flat even ground at a good pace, but if the ground is uneven or there are hills or stairs I need to use a walking stick and I must slow right down and look down which can cause problems with the Cervical Dystonia and breathing issues. I am not

able to play any physical sports. I I spend a lot of time down the South Coast watching the whales from my home which is very relaxing and peaceful. I also love walking on the beach but walking in the dry sand can be quite tiring.

Q 31 – Rate the importance of each activity in managing your CD

Activity	Very important	Somewhat important	Not important	Total
Medication	72	19	15	106
Exercise	43	39	12	94
Rest / sleep	79	19	2	100
Healthy diet	55	29	8	92
Meditation / Mindfulness	27	27	22	76
Social interactions with family / friends	45	38	13	96
Continuing Hobbies	53	30	14	97
Support of family / friends	65	26	6	97
Support of others with dystonia	43	28	17	88

Final comments:

I have had to completely manage my dystonia. There do not appear to be any experts in the field. Each person I see tells me there is no really effective treatment and it’s a matter of trial and error. No neurologist I have seen has ever suggested I see any other therapist. It seems to be generally accepted that because focal dystonia is comparatively rare there is little research into the condition. Professionals in the movement disorder appear to prefer to specialise in the more common movement disorders such Parkinson’s disease where they can see many more patients and earn more income.

My distonia has been occurring for so long and I have tried so many different treatments that don't make a difference or make the trembles worse that I just try to live my life the best I can with the distonia and do what I can to help myself depending on the reasons the condition either abates a little, or gets worse at certain times

No thank you

Last 2 months I have done 3 standing yoga stretches every morning and have noticed a big reduction in the frequency of headaches I normally experience. I attribute the headaches to

overuse of shoulder & neck muscles that have been weakened by botox*. Headaches normally start overnight and come from pain in back of my skull and neck.

Overall, I find the best way to manage my pain is to keep occupied. I am retired and find gardening, hobbies etc keep me occupied. This helps greatly to take my mind off the pain.

My speech therapist was the health care staff member who took notice of my cervical dystonia. My vocal dysphonia was my main problem and my concern used to be that I was developing more other sorts of dystonia (blepherospasm). My kidney function continued to deteriorate until I was put on peritoneal dialysis and then had a kidney transplant. Dystonia became of less concern.g

Two things: I manage where I sit and stand in relation to others when I interact with them, and where I sit in an audience. I find high speed driving stressful, especially highway lane changes, and avoid these. Also avoid driving more than one hour, as this is painful. I tell the people who have to know what my challenges are, then don't focus on them, and get on with the busyness of work and life. I can be more aware of pain and spasms when I rest at the end of the day.

It is really important to be able to talk to fellow sufferers.

My cervical dystonia impacts my levels of enjoyment of life and ability however I work hard at balancing this out and not getting scared or worried to much. I find botox* injections useful but also very overwhelming.

I just had to realise I couldn't achieve all I wanted and find other things I could do. It was easier to become self employed

Just wish the tremors would go away

I found that intense phisio or masage on my neck made it much worse and it stayed worse. However I have found a physio that has been able to release the tension in my neck with very minimal manipulation and this is very helpful to give better movement. I also found that wearing a loose neck brace in the office gave my chin a spot to locate and after many months I dont need the brace anymore and can find the spot and feel stable with it. This effect has stayed for 3 years and not changed. Excercise makes the dystonia play up but is more reduced the next day it is always worse when I am doing nothing and am not looking after myself. Swimming can be difficult to breath to one side but I am hoping I can learn this to be a movement that has a reduced dystonia effect. I have purchased lounges with headrests at the right height to watch tv and relax at home and this will be where I sit to relax myself and turn down the dystonia effect if it is playing up.

I feel I do the very best I can. Apart from my neurologist I see no one related to my Dystonia. My Doctor has limited understanding I feel. I also feel that I do not know very much about my own condition either. It varies so much with each individual. I am fortunate in having many good days but the bad days are becoming more frequent and more prolonged. This is where I feel information is lacking for me.

My neurologist has control of medical management for my dystonia but I ensure that I supply a consistent, updated symptom diary that is discussed every 3 months and used, alongside my consultation presentation, to underpin the botox* therapy approach. My neurologist is open to my suggestions of alternatives to try

The key element for me is to have as much information as possible when you are diagnosed and a plan

I haven’t tried all if the options you have mentioned. I’m unaware of them

I feel like ive been bumbling along trying to manage with very limited information and no real outside Professional medicAl help. This is very exhauting. I dont get alot of sleep. p

CD is a miserable existence its phycological warfare. The greatest battle is not the physical it’s keeping your head in the game day to day. CD never leaves you its just bad a little bad or a lot bad.

Use a walking stick as reassurance for balance. Sit forward on chairs. Take 10mg of diazapem before bed.

I manage it on a day to to day basis and it's best not to plan too far in advance. I have to resolve myself that it is OK to take pain relief in greater doses when needed.

Mental health is important to help reduce stress levels. Working on breathing through meditation and staying active. I have a massage every 1-2 weeks to help reduce tightness leading to other potential issues. I try and eat well to stay healthy. Looking after myself in every other war possible

I feel as though participating in previous research has helped me learn the sorts of relaxation, meditation and gentle exercise which helps me manage my cervical dystonia, so thank you for specialising in my area of need.

Everything I have tried only gives temporary relief from spasms and pain

It is a very variable condition so getting managemnet right can be difficult. Very tiring & i guess misunderstood condition. It is & has been a long road. Currently on Sick leave as i wouldn't & choose not to put myself under the extra stress of my job. Difficult to put one answer or be able to answer the survey well.

I have foudn the best way to manage my dystonia is to listen to my body and act accordingly. Whilst medication and Botox* allow me to function day to day, I still have to manage myself. I am self employed and that allows me to stop when I need to. I had to leave the job I love because I could not keep up with it. Everything in my life is revolved around what I can physically manage. I work full time but I do it at my pace. Until you learn to listen to your body and do things at your own pace, you will always struggle more and therefore become less productive over all. Sometimes the only way i can get relief is to lie on my left side. My left side is the my dystonia side. Weight also helps. Sometimes I lie on my left side and get my husband or kids to lie on top of me because the weight stops the spasms and eases the cramping,

My physio Group Rehab Class (circuit) is very important to me as I can concentrate on the rest of my body which in turn helps with my neck.

I try not to focus on my CD so that coping strategies become more like a sub routine in my daily activities.

It's best when I don't focus on dystonia and put it in the back of my mind. Avoiding activities that aggravate my dystonia helps a lot (e.g. extended computer use, strenuous activity, use of upper body, twisting my head in a difficult direction, uncomfortable chairs). Living my life using only slow gentle movements helps. Accepting I have a disability helps. Finding what there is left to enjoy helps.

It’s very difficult to manage and live with CD.There isn’t enough support and knowledge about it. They don’t even know what causes it.

No. Thank you for doing this survey, Melani.

Surgery was my answer although too late to save my career

It’s confronting when first diagnosed. It’s up to you to search for information and treatments, which is why support groups are critical. The dearth of treatments is hard. Reducing stress to a minimum is key to managing the condition and remaining stable. It makes working or many activities are out of bounds.

Massage, sports and stretching is great, but only provides short-term relief

Thank you Melani for your research!

Maybe more groups of people with it to interact and talk to about it

no

I think for me it is most important to continue with physio and massage. If I miss a week my body certainly tells me. My reason for being so adamant about physio and massage is with Dystonia it is important to keep the muscles relaxed espiecially around the area of Dystonia. Cervical Dystonia dosent only affect the neck but when you shake or co tract the shoulder blade muscles,mid back ,lower back all hold tension. Reliving this I think helps me cope with my Dystonia and relieves the headaches. My neuro would like me to have DBS but we live rural and I have to get my kids to bus stop for school etc.. Dystonia it not really recognised by Centrelink or ndis which is a shame as it’s debilitating. Not only do we suffer pain but we suffer unwanted comments from outsiders.I know that I couldn’t live without my Therapists,they make a huge difference allowing me to live a partly normal life

心理也很重要，需要多放松冥想和休息 - Psychology is also very important, and it is necessary to relax and meditate and rest more

CD for me manifests as a head tremor. Apart from an inability to turn my head to the right I have no pain but a very visible tremor. This affects my social life severely as I am very aware of it and feel very self conscious when in public. I was diagnosed with CD 20 + years ago but my tremor has only become much more noticeable in the last few years. Tiredness and

stress make it worse. I find a glass of alcohol (which I dislike..) causes the tremor to improve considerably so I do use it as a coping strategy occasionally. I also hold my head (geste?) when sitting upright say at a dining table and that relieves the tremor. I have hypertension and have recently had my daily medication doubled (Propranolol 160mg) which I feel has helped my tremor a lot. Hope this helps your studies.

At 5 years swimming changed my life + yoga in the last 4 years improved things enormously

I find my condition extremely debilitating & frustratingly difficult to live with.

Management and treatment of depression.

I feel very alone in managing my Cervical Dystonia. My GP doesn't understand it and can't offer me advice. It would be good if a number of GP's in each State specialised in Cervical Dystonia (and other dystonias). There is no coordinated treatment. There is only one neurologist in WA that does 'guided' Botox* injections (the others don't feel it necessary despite poor outcome). I feel like I've been in the medical gutter for twenty years. It is soul destroying, but on the upside I have learned how strong and resilient I am, developed deep empathy for others in chronic pain, and gained a lot of knowledge of anatomy and movement disorders generally and their treatment through reading.

Spasms are few and far between but what helps me most to overcome them is sleep. I liken the symptoms to migraines which I had before my neck started to tremor.

It took more than 5 years to be diagnosed after multiple trips to different doctors and mri scans. Unfortunately, the level of understanding amongst GPs is close to nonexistent, in my experience.

minimizing stress and more sympathetic employers

Seeing a new doc at moment , more people nèd to ge made aware of it

We need to find what works for us well. With Dystonia i was not able to wash face but if i look down and wash face in basin then its works well. When i am riding motorcycle or bike, i don't feel any paid while turning a neck. So i prefer riding bike a lot.

I cannot wear cross body bags or back packs. I am unable to tolerate necklaces except very light ones.

The affordability of Botox* injections are difficulty due to the cost of the neurologist but the cost of the Boto*x itself is manageable. My quality of life and difficulty stems from pushing through life requirements without support - for example ironing or loading of dishwasher are difficult. Sweeping or vacuuming often result in migraine or muscle spasms

More consistency, more options. It’s very much trial and error, and every neuro has a different opinion.

I am so pleased that you are doing your PHD on Cervical Dystonia. It is one of many unknown illnesses that no one has heard of. It needs to get all the attention it can, so that people know where to go to find help.

HOW TO COPE WITH TORTICOLLIS WHICH IS HELL, LESS HELL THAN DYSTONIA. SINCE DYSTONIA, I CANNOT HAVE TEA OR SOUP. COULD WHEN I LIVED ON THE GOLD COAST BEFORE I CONTRACTED NEUROMUSCULAR DYSTONIA. DYSTONIA AND FIBROMYALGIA WERE DIAGNOSED BY DR. DARYL SALMON, DIRECTOR OF CRITICAL CARE AT LIVERPOOL HOSPITAL.

I find managing my stress to be very helpful. I've always been a worrier. Stress tenses up my body a lot which affects the Dystonia symptoms on any given day. I also find the position in which I sleep affects it too which is something I haven't been able to get to the bottom of. I feel like it's a very big clue. thank you for working on this. Leah

Learning yoga was my biggest breakthrough in managing my CD

I have found Ultra Muscleze Magnesium Cream to be very beneficial for helping with sleep as the dystonia stops me from relaxing my neck.

There is nothing else that I use to manage my dystonia because it keeps changing and even Botox* injections don't always work. Support from other members of the Australian Dystonia Support Group - a private Facebook page.

There is very little information out there on how to mange CD. My GP hadn't even heard of it before me. I feel quiet vulnerable knowing I am responsible for my treatment. Botox* is so hit and miss. Its up to the skill of the Dr which muscles he injects. I would like to see more exercise programs in Australia to help with this condition.

I have learnt what stimulus makes it worse eg crossbody bag, heavy jewelry and heavy lifting

My cervical dystonia was very severe in the first few years but after a self managed diet and exercise routine went into full remission. I now have intermittent spasms and pain which is manageable.

I have become acclimatised but not accepting. I find funny memes help "normalise" the condition eg on dystopia Europe

With patience and an attitude that things are getting better every day

Make sure you have a good Neurologist. Once they get your injection pattern correct then you will see a great improvement.

Dr Joaquin Farias' Dystonia Recovery Programme is working the best for me. You have to be very committed to doing all the exercises and following the protocol but it works

Health care is fragmented. I would like to see more coordination between health professionals to manage treatment

Extended social interaction aggravates pain and spasm, as does walking. I walk daily but tend to avoid social situations

I have had to learn other ways to do things eg eating; carrying things; writing, applying makeup

It gets lonely sometimes having to deal with CD as my family/ friends/ dystonia support group are great but it would be a comfort to know that somewhere a health professional was looking out for me. I may be an expert in how my dystonia affects me and what can ease the worst of the symptoms but the input of a movement disorder specialist or health professional would really benefit me and enhance my quality of life.

this is a very limited survey..i assume it takes in Spasmodic Dysphonia??? The use of CBD Decarb has been the most important help
